# *CTLA4* Alteration and Neurologic Manifestations: A New Family with Large Phenotypic Variability and Literature Review

**DOI:** 10.3390/genes16030306

**Published:** 2025-03-03

**Authors:** Edoardo Genio, Mauro Lecca, Rachele Ciccocioppo, Edoardo Errichiello

**Affiliations:** 1 Unit of Medical Genetics, Department of Molecular Medicine, University of Pavia, 27100 Pavia, Italy; edoardo.genio01@universitadipavia.it (E.G.); mauro.lecca01@universitadipavia.it (M.L.); 2 Gastroenterology and Endoscopic Unit, Department of Medicine and Ageing, University Gabriele D’Annunzio of Chieti-Pescara, 66100 Chieti, Italy; rachele.ciccocioppo@unich.it; 3 IRCCS Mondino Foundation, 27100 Pavia, Italy

**Keywords:** *CTLA4*, haploinsufficiency, immunodeficiency, autoimmunity, demyelination

## Abstract

Cytotoxic-T-lymphocyte-antigen-4 (CTLA-4), a member of the immunoglobulin superfamily, is an essential negative regulator of immune responses that is constitutively expressed on both regulatory (Treg) and activated T cells. To date, heterozygous germline variants in *CTLA4*, leading to haploinsufficiency, have been associated with several immunological disorders, including hypogammaglobulinemia, multi-organ autoimmunity, lymphoproliferative disorders, and enlarged lymphoid organs. Indeed, *CTLA4* carriers display highly heterogeneous clinical manifestations with a phenotypic spectrum ranging from asymptomatic carrier status to fatal autoimmunity. Here, we describe a family with autoimmune phenotypes (Hashimoto thyroiditis, psoriasiform dermatitis, celiac disease/inflammatory bowel disease, and rheumatoid arthritis), segregating across three different generations due to a recurrent missense variant [c.436G>A, p.(Gly146Arg)] in the *CTLA4* gene. Interestingly, the proband showed prominent neurological manifestations, including seizures, hydrocephalus, and demyelination, which are less frequently reported in individuals with pathogenic variants in *CTLA4*. A detailed literature review of neurologic features that have been reported so far in *CTLA4* carriers is also provided.

## 1. Introduction

*CTLA4* (MIM *123890) encodes the cytotoxic T-lymphocyte antigen 4 (CTLA-4, also known as CD152), a transmembrane protein constitutively expressed on the surface of regulatory T cells (Treg) and activated T cells. It negatively modulates T cell activation and contributes to peripheral tolerance by competing with the stimulatory molecule CD28 for binding to CD80 and CD86 ligands on the surface of APCs (antigen-presenting cells) [1].

Homozygous variants of *Ctla4* have been associated with fatal multi-organ lymphocytic infiltration and destruction in mice [2,3]. Biallelic variants have never been described in humans thus far, whereas pathogenic heterozygous variants, often showing incomplete penetrance and variable expression, are related to a rare primary immunodeficiency (prevalence <1/1,000,000; ORPHA:436159); this is characterized by a variable combination of enteropathy, hypogammaglobulinemia, recurrent respiratory infections, granulomatous lymphocytic interstitial lung disease, lymphocytic infiltration of non-lymphoid organs (i.e., intestine, lung, brain, bone marrow, and kidney), autoimmune thrombocytopenia or neutropenia, autoimmune hemolytic anemia, and lymphadenopathy [4,5,6,7]. Loss of function/haploinsufficiency is considered the main pathogenic mechanism, as reflected by gene predictive scores (pLI:1.00; LOEUF:0.21), although negative dominance has also been hypothesized [8]. In the Human Gene Mutation Database (HGMD; last access 18 February 2025) there are 140 *CTLA4* variants listed (114 in coding and 26 in non-coding regions), with missense/nonsense, deletions, and splicing substitutions representing the most frequent classes of genetic alterations (72, 26 and 12 variants, respectively).

In a large cohort of *CTLA4* carriers, neurologic manifestations were reported in about one third of individuals, mainly encephalitis/encephalomyelitis, seizures, headache, and nausea [6]. Here, we describe a three-generation family showing highly heterogeneous clinical phenotypes, including neurologic manifestations, caused by the recurrent c.436G>A (p.Gly146Arg) missense variant in *CTLA4*. A detailed literature review of neurologic phenotypes reported in *CTLA4* carriers is also provided.

## 2. Subjects and Methods

### 2.1. Clinical Phenotype

**Figure 1:**
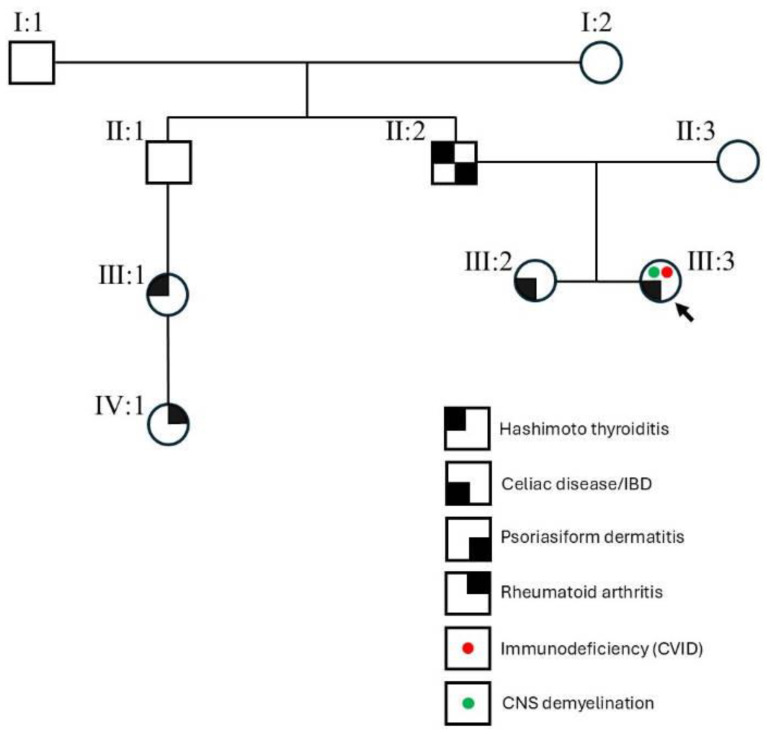
Family pedigree showing variable immune-mediated phenotypes associated with the pathogenic heterozygous missense variant c.436G>A (p.Gly146Arg) in *CTLA4*.

The proband was a 45-year-old female born to non-consanguineous Italian parents. At 33 years of age, she came to our attention due to chronic diarrhea. Her family history was suggestive of autoimmune disorders, namely Hashimoto thyroiditis, psoriasiform dermatitis, celiac disease, and rheumatoid arthritis (Figure 1). At the age of 13, the proband was diagnosed with common variable immunodeficiency (CVID). Clinical records documented lymphopenia, postnatal growth retardation, interstitial pneumopathy and bronchiectasis (with a moderately decreased DLCO%), recurrent sinusitis, and other severe opportunistic infections. Six years later, she underwent treatment with cyclosporin, steroids, infliximab, and immunoglobulins, following a diagnosis of hemolytic anemia and autoimmune thrombocytopenia, along with a modest erythroid dysplasia diagnosed by bone marrow biopsy. At the age of 21, the proband was admitted to hospital following episodes of loss of consciousness. Head CT (Computed Tomography) highlighted pons hypodensity, likely suggestive of an ischemic stroke. One year later, she was admitted for an epileptic episode triggered by fever. At the age of 23, the proband underwent a splenectomy because of thrombocytopenia, and a cholecystectomy due to acute cholelithiasis. Afterwards, she began to manifest recurrent muco-hematic diarrheic episodes with dehydration; however, the diagnosis was uncertain (either inflammatory bowel disease (IBD), infective colitis, or autoimmune colitis.) At age 28 years, a brain MRI showed focal lesions of the vermis and left hemisphere, which were interpreted as autoimmune-related demyelination, and supratentorial hydrocephalus; this was treated with a ventriculoperitoneal shunt (VPS). The latest endoscopic follow-up revealed a colorectal polyposis. Laboratory investigations also confirmed hypogammaglobulinemia with almost undetectable values of IgA (<6 mg/dL; n.v.: 40–350 mg/dL), IgE (<2 kU/l; n.v.: 0–380 kIU/L), and IgM (5 mg/dL; n.v.: 54–300 mg/dL), but normal IgG (664 mg/dl; n.v.: 650–1600 mg/dL for adults) because of scheduled IgG therapeutic infusions. Further immunological assessments showed an almost complete absence of B lymphocytes (0.6%, n.v.>6%), reduced T cell proliferation in vitro, and significantly decreased circulating FOXP3+ regulatory T (Treg) cells. Anti-MuSK (Muscle-Specific Kinase) and anti-AChR (Acetylcholine Receptor) antibody tests were negative, as was a cerebrospinal fluid (CSF) analysis, an evoked potentials test, and other blood tests to rule out other conditions, such as infections (e.g., Lyme disease, syphilis, HIV, and Parvovirus), vitamin deficiencies (e.g., B12), and autoimmune diseases. Osteoporosis (t-score −2.8, z-score: −2.7) was also found by means of bone mineralometry.

The study was conducted in accordance with the local legislation and institutional requirements. Written informed consent for genetic analysis and publication of the clinical data was obtained from the participating subjects.

### 2.2. Genetic Analysis and In Silico Predictions

Whole-exome sequencing (WES) was performed on the proband and parental DNA samples (trio-WES), extracted from peripheral blood by standard procedures, using the Human Core Exome kit (Twist Bioscience, South San Francisco, CA, USA) on a NovaSeq 6000 system (Illumina, San Diego, CA, USA). The sequencing reads were aligned against the human reference genome (GRCh38/hg38). Variant calling was performed according to international guidelines using an in-house developed pipeline. In brief, the bioinformatic analysis was focused on variants with a frequency <5% in gnomAD v4.1.0 and an in-house database (~9500 samples), focusing on a virtual panel of 564 genes related to primary immunodeficiency and inflammatory bowel disease (PanelApp version 7.21). Variants were classified according to the ACMG-AMP and ACGS guidelines https://www.acgs.uk.com/quality/best-practice-guidelines/ accessed on 29 January 2025 and specific ClinGen recommendations (https://clinicalgenome.org/working-groups/sequence-variant-interpretation/) (last access 29 January 2025) [9]. The variant nomenclature was verified using Mutalyzer (https://mutalyzer.nl/ accessed on 29 January 2025) and VariantValidator (https://variantvalidator.org/ accessed on 29 January 2025).

Copy number variants (CNVs) were detected with Control-FREEC and EXCAVATOR tools [10,11]. Sanger sequencing was used for segregation analysis in the available family members and to search for known modifying/regulatory variants, particularly those located in non-coding regions uncovered by ES experiments, that could explain the observed intrafamilial phenotypic variability. A three-dimensional (3D) model of mutated protein structure was predicted using AlphaFold (https://alphafold.ebi.ac.uk/ accessed on 29 January 2025) and visualized with PyMOL 3.1 (https://pymol.org/ accessed on 29 January 2025). I-Mutant2.0 (https://folding.biofold.org/i-mutant/i-mutant2.0.html accessed on 29 January 2025) was used to predict the effect of the variant on protein stability. STRING v12.0 (https://string-db.org/ accessed on 29 January 2025) and Cytoscape v3.10.3 (https://cytoscape.org/ accessed on 29 January 2025) were used to investigate CTLA-4 interaction networks.

## 3. Results

### 3.1. Genetic Findings

Trio-WES identified a pathogenic heterozygous missense variant within exon 2 of *CTLA4* (NM_005214.5:c.436G>A; p.Gly146Arg). The variant is reported in ClinVar (VCV000849622.11), HGMD (CM164030), and PubMed, and falls in a mutational hot-spot along with two other disease-causing variants (c.436G>T and c.437G>T) [6,12,13,14,15,16,17,18,19]. The variant was inherited from the affected father and co-segregated with the disease in the family (Figure 1).

The Gly146Arg variant in CTLA-4 is located within the ligand-binding domain [12], which includes the highly conserved ‘MYPPPY’ motif (aa 134–139). This motif is crucial for interactions with CD80 and CD86 and represents a mutational hot-spot, containing about one third of the disease-causing variants [20]. The missense change replaces glycine, which is neutral and non-polar, with arginine, which is basic and positively charged. A comparison between wild-type and mutant structures revealed significant 3D changes (RMSD = 2.18). GRAVY index scores of hydropathy (WT: 0.296; MUT: 0.278), indicated lower hydrophobicity of the mutant protein, while I-Mutant2.0 returned a negative ΔΔG value (−1.01 Kcal/mol), consistent with a loss of protein stability (Supplementary Figure S1). These in silico predictions are in line with previous functional studies showing significantly decreased levels of CTLA-4 protein in T cells of c.436G>A carriers compared with wild-type individuals [13,15].

To further investigate the observed intrafamilial variability, we searched for common *CTLA4* variants that have been previously reported to modify penetrance, namely rs3087243 (CT60) and rs231775 (CTLA4+49); these are located within the 3′-UTR and first exon of *CTLA4*, respectively. The proband and an affected sister showed a G/G genotype for CT60, associated with a low expression of a soluble isoform of CTLA-4 lacking the third exon and a predisposition to autoimmune diseases, whereas the father was heterozygous A/G [21,22,23,24,25]. On the other hand, an A/A CTLA4+49 genotype was observed in all the affected family members. Because the inheritance pattern of such *CTLA4* variants in the pedigree did not consistently explain the observed intrafamilial phenotypic variability, we searched for other potential genetic modifiers in genes encoding CTLA-4 interactors (Supplementary Table S1), under the hypothesis that additional ‘hits’ may worsen the proband’s clinical presentation. The only candidate variant, NM_175862.5:c.15-81C>A (rs915120854), detected in the proband but not in the relatives with milder clinical manifestations, was located in intron 1 of *CD86*. *CD86* encodes the functional ligand of CTLA-4, which is required for inhibitory signals to prevent CD28-mediated T cell activation [26].

### 3.2. Phenotypic Constellation in CTLA4 c.436G>A Carriers

Neurologic and neuroradiologic features are described in a minor and variable portion of *CTLA4* carriers. In a large study where 133 individuals were enrolled, a broad spectrum of neurologic manifestations, ranging from nausea and headache to aphasia and encephalitis, were reported in 25 of them (~19%) [6].

According to HGMD, the c.436G>A variant (CM164030) detected in our family has been reported in only 10 other individuals (Table 1). Gastrointestinal features, including Crohn’s disease, gastritis, and enteropathy, were reported in 8 out of 10 subjects; respiratory anomalies, including pneumopathy and recurrent pulmonary infections, in 6/10; hematological alterations, including autoimmune hemolytic anemia, iron deficiency anemia, immune thrombocytopenic purpura, and Evans syndrome, in 6/10; skin and skeletal anomalies in 4/10 each; endocrine manifestations (type 1 diabetes) in 2/10; renal involvement was observed in 1/10. Neurologic symptoms were detected in four of the subjects, including recurrent paralysis of limbs, abnormality of vision, anxiety, seizures, recurrent headache, paraparesis, nausea, and fecal incontinence (Table 1); one showed intramedullary lesions on a brain MRI scan.

Our proband showed seizures, hydrocephalus, vermis lesions, and demyelination as the main neurological/neuroradiological symptoms. As far as we know, such neuroradiological lesions have never been reported in *CTLA4* heterozygotes. We ruled out the presence of other disease-causing variants in genes associated with brain malformations, especially hydrocephalus, vermian anomalies and demyelination, and epilepsy, that might be involved in the neurophenotype observed in the proband. 

## 4. Discussion

CTLA-4 is a key regulator of immune homeostasis whose alterations have been observed in many autoimmune disorders. The clinical history of our family emphasizes the broad phenotypic variability and incomplete penetrance associated with *CTLA4* variants, estimated to be between 55% and 70% [6,8,27]. To the best of our knowledge, such a large intrafamilial variability has never been reported before, at least for the c.436G>A variant, and underlines the pleiotropy of *CTLA4*.

The extremely variable spectrum of neurologic characteristics reported in up to 30% of *CTLA4* carriers from childhood to late adulthood, includes headache and seizures (as leading symptoms), encephalitis/encephalomyelitis with cerebral perivascular lymphocytic infiltration, nausea, aphasia, visual impairment, ventriculomegaly, and slowly progressive cognitive deterioration (Table 1) [5,14,15,28,29,30]. Our proband showed lesions of the cerebellar vermis, seizures, hydrocephaly, and demyelination. The latter is commonly observed in multiple sclerosis (MS), where *CTLA4* downregulation has also been reported, while certain polymorphic variants in *CTLA4* are linked to reduced remyelination in MS, suggesting potential common underlying immunologic mechanisms between *CTLA4* deficiency and ‘genuine’ MS [31,32,33,34,35,36]. Interestingly, tumefactive demyelinating lesions on a brain MRI that were suggestive of MS have been reported previously in a 14-year-old girl carrying a different pathogenic *CTLA4* variant (c.208C>T, p.(Arg70Trp)) [37]. *CTLA4* alterations have also been associated with an increased susceptibility to myasthenia gravis (MG). Further, functionally abnormal Treg cells have been found in MG individuals with a low expression of *CTLA4* [38,39]. In a previous cross-sectional observational study, cerebellar lesions were identified in 75% of *CTLA4* subjects, while demyelination was reported in a 48-year-old female proband carrying a heterozygous frameshift variant in *CTLA4* exon 1 (c.81dup; p.Leu28Serfs*32) [40,41]. Several systemic autoimmune disorders, such as systemic lupus erythematosus, display hydrocephaly as a symptom, likely as the result of direct damage to the small-sized venous structures, or immune complex deposition within the arachnoid villi leading to a lack of reabsorption of the cerebral spinal fluid [42]. The emergence of these lesions in *CTLA4* carriers can be explained by the lack of CTLA4-mediated inhibitory signals for T cell activation, caused by an altered ability of CTLA-4 to bind the costimulatory ligands CD80 and CD86. This causes T cell infiltration of the brain and loss of blood-brain barrier integrity, which in turn induces a state of persistent neuroinflammation leading to demyelination, periventricular, cerebellar, and spinal cord injury.

Interestingly, the neurological manifestations observed in the proband, especially demyelination, recapitulate some of the side effects of cancer immunotherapy using immune checkpoint inhibitors (ICIs), such as the CTLA4-blocking monoclonal antibodies ipilimumab and tremelimumab [43,44,45,46,47,48,49]. This evidence suggests caution when using ICIs in *CTLA4* carriers, as well as the potential risk of administering ICIs to subjects with clinically silent *CTLA4* haploinsufficiency.

In conclusion, neurologic involvement may be underestimated in *CTLA4* deficiency because of the predominant extra-neurologic features. However, neurologic manifestations should be taken into consideration in the diagnostic work-up of subjects carrying symptoms commonly related to *CTLA4* haploinsufficiency, such as CVID and multi-organ autoimmunity. Referring patients with familial opportunistic infections and organ autoimmunity to tertiary centers where appropriate diagnostic procedures are available, together with their management by a multidisciplinary team (including immunologists, clinical geneticists, hematologists, neurologists, and gastroenterologists, as appropriate) is strongly recommended to achieve optimal management and improve their clinical outcome. In these cases, immunophenotyping and a genetic assessment may be very helpful in reaching a definitive diagnosis. This is because a potentially effective targeted immunotherapy, such as abatacept (a fusion protein consisting of the extracellular domain of human CTLA-4 bound to the modified Fc portion of human IgG1, which selectively modulates a key costimulatory signal required for the full activation of CD28-expressing T cells), or allogeneic bone marrow transplantation [50], may be offered to affected subjects.

## Figures and Tables

**Table 1:** Phenotypic manifestations associated with the *CTLA4* c.436G>A variant.

Study (PMID)	Immunological	Gastrointestinal	Respiratory	Neurological	Hematological	Skin	Endocrine	Skeletal	Others
29729943 (Individual #53, fam V.II.1)	Lymphadenopathy	Lymphocytic organ infiltration (retroperitoneum)			ITP; AIHA; autoimmune neutropenia; Evans syndrome				Renal involvement
29729943 (Individual #99, fam SS.II.1)	Hypogammaglobulinemia (low IgG, IgM, IgA); fungal infections (Candida)	Splenomegaly; diarrhea/enteropathy, atrophic gastritis	Respiratory involvement, (upper and lower respiratory tract); severe respiratory infections (pneumonia); GLILD; bronchiectasis; lymphadenopathy and lymphocytic organ infiltration (lungs)	Lymphadenopathy and lymphocytic organ infiltration (brain); retinal tear due to lymphocytic infiltrations into the retina				RA	
28960754	Autoimmune lymphoproliferative syndrome (ALPS)	Hepatomegaly; splenomegaly			Evans syndrome				
31993940	Positivity for anti-parietal cell antibody; hypogammaglobulinemia (low IgG)	Recurrent diarrhea; atrophic gastritis; lymphoid hyperplasia at the ileal end	Recurrent respiratory infection	Dysphagia since age 3 years	IDA; ITP; leukopenia;	Bilateral axillary lymphadenopathy		Cervical lymphadenopathy; bilateral knee arthralgia; steroid refractory RA	
32996901	Reduction of CD19+ B cells, low levels of total IgG and IgA, and a normal IgE level; CMV infection; reduced PBMC count	Chronic diarrhea; enteropathy; cholecystectomy (probably due to AIHA)	Chronic sinusitis		ITP; Evans syndrome	Alopecia areata	Hypothyroidism, diabetes mellitus type 1		
35753512 (P0003698)	Chronic infections; fever	Abdominal pain; splenomegaly; diarrhea	Abnormal lung morphology; pulmonary obstruction	Abnormality of vision; nausea; vomiting; anxiety; seizures; abdominal pain; distal muscle weakness; headache	AIHA; IDA	Urticaria; blepharitis			Weight loss
32623363	Hypogammaglobulinemia; polyvalent allergy	Crohn’s disease; sphincter dysfunction (bowel)	Bilateral pulmonary interstitial infiltration; chronic pansinusitis	Progressive headache and focal right-side-sensitive epileptic paroxysm; presence of an isolated infiltrating mass (16 × 20 × 22 mm) and lesions in the white-matter (left parieto-occipital region); T2-hyperintense lesion in the right counterpart; positive sensory symptoms in both hands; static tremor and hypesthesia of the left upper extremity (suspected MS); idiopathic intracranial hypertension; moderate central paraparesis of the lower extremities; hypesthesia; disseminated intramedullary lesions; CSF oligoclonal bands			Autoimmune thyroiditis	Juvenile seronegative RA	Recurrent uveitis, with permanent moderate vision loss due to papilledema; sphincter dysfunction (bladder)
31955317	Immunodeficiency with low IgG and undetectable IgA and IgM levels; CD19+ B cells were absent; lower count of CD4+ T cells; elevated lactate dehydrogenase level	Cholestasis; mediastinal adenopathy; hepato-splenomegaly, multiple lytic lesions of the liver	Interstitial fibrosis of the left lung with focal honeycombing and lymphocytes infiltrates in the fibrotic areas; sinopulmonary infections; progressive respiratory distress with hypoxemia; dyspnea		ITP progressed to pancytopenia with lymphopenia by age 23 years; erythroid and megakaryocytic hyperplasia	Anasarca; axillary lymphadenopathy		Multiple lytic lesions in the skull, ribs, and spine; cervical lymphadenopathy	
36790564	Unspecified primary B cell defects								
30443250	Immune dysregulation-IPEX								
Current study	Immunodeficiency; hypogammaglobulinemia (low IgG, IgA, IgM, IgE); CVID	Chronic diarrhea; enteropathy; gallstones; celiac disease/IBD; poliposis	Interstitial pneumopathy	Hypodensity in the pons region; vermis lesions; hydrocephalus; seizures; CNS demyelination	Autoimmune thrombocytopenia; hemolytic anemia	Psoriasiform dermatitis (father)	Hashimoto thyroiditis (father and cousin)	Osteoporosis; RA (niece)	Growth delay

AIHA (Autoimmune Hemolytic Anemia), ALPS (Autoimmune Lymphoproliferative Syndrome), BCG (Bacillus Calmette–Guérin), CSF (Cerebro-Spinal Fluid), CT (Computer Tomography), CVID (Common Variable Immuno-Deficiency), GLILD (Granulomatous Lymphocytic Interstitial Lung Disease), IBD (Inflammatory Bowel Disease); IDA (Iron Deficiency Anemia); IPEX (Immune Dysregulation, Polyendocrinopathy, Enteropathy, X-Linked), ITP (Immune Thrombocytopenic Purpura); MS (Multiple Sclerosis); PBMC (Peripheral Blood Mononuclear Cells); RA (Rheumatoid Arthritis).

## Data Availability

The data that support the findings of this study are available on request from the corresponding author. The data are not publicly available due to privacy or ethical restrictions.

## Supplementary Materials

The following supporting information can be downloaded at: https://www.mdpi.com/article/10.3390/genes16030306/s1, Figure S1: Hydropathy plots (Expasy ProtScale) comparing wild-type (left) and mutant (right) CTLA-4; Table S1: List of CTLA-4 interactors;
