# Supplementary material for: CTLA4 Alteration and Neurologic Manifestations: A New Family with Large Phenotypic Variability and Literature Review
Source: Genes (Basel). 2025 Mar 3;16(3):306. doi: 10.3390/genes16030306 (PMC11942126; doi:10.3390/genes16030306)
Supplement: Supplementary file 1 [file genes-16-00306-s001.zip › genes-3480155-supplementary.pdf]

Supplementary Figure S1

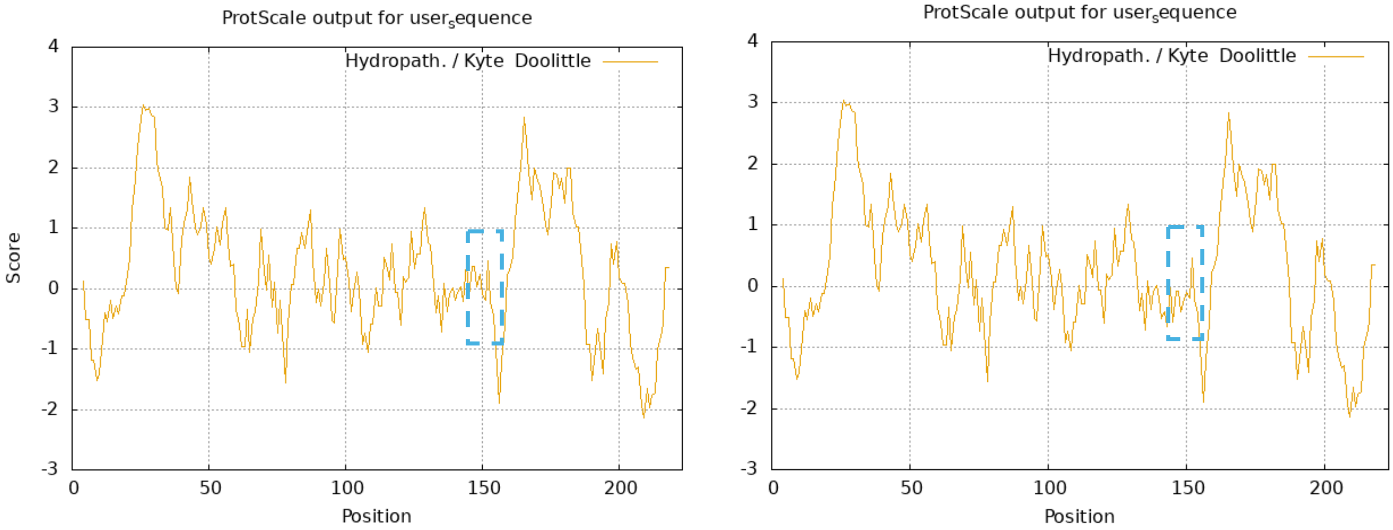

Figure S1. Hydropathy plots (Expasy ProtScale) comparing wild-type (left) and mutant (right) CTLA-4. The Gly>Arg change at position 146 of CTLA-4 (blue box) modifies the hydropathy properties of the mutant protein by decreasing hydrophobicity.

Supplementary Table S1

Table S1.

List of CTLA-4 interactors
CCR7, CD160, CD27, CD274, CD276, CD28, CD40, CD40LG, CD58, CD70, CD80, CD86, CXCR5, FOXP3, HAVCR2, HHLA2, ICOS, ICOSLG, IDO1, IDO2, IGHV3-43D, ITGAE, KLRC1, KLRG1, LAG3, LGALS9, LGALS9B, LGALS9C, MTUS1, MTUS2, NCR3LG1, NECTIN2, PDCD1, PDCD1LG2, PVR, SLC44A4, TBX21, TIGIT, TMIGD2, TNFRSF14, TNFRSF18, TNFRSF4, TNFRSF9, TNFSF18, TNFSF4, TNFSF9, VSIR, VTCN1
